# Healthy Ageing in People with Intellectual Disabilities from Managers’ Perspective: A Qualitative Study

**DOI:** 10.3390/healthcare5030045

**Published:** 2017-08-18

**Authors:** Maria Johansson, Petra Björne, Ingrid Runesson, Gerd Ahlström

**Affiliations:** 1 Department of Health Sciences, Faculty of Medicine, Lund University, SE-221 00 Lund, Sweden; maria.johansson@med.lu.se; 2 Research and Development Unit, City of Malmö, SE-205 80 Malmö, Sweden; petra.bjorne@malmo.se; 3 Department of Social Work, Faculty of Health and Society, Malmö University, SE-205 06 Malmö, Sweden; ingrid.runesson@mah.se

**Keywords:** active ageing, content analysis, daily activity centre, group home, healthy ageing, intellectual disability, mental retardation, older people, qualitative interviews, successful ageing

## Abstract

An increasing number of people with intellectual disability (ID) are reaching older ages today although they experience more health problems than the older population without ID. Leaders in intellectual disability services can greatly influence the conditions for a healthy ageing, and the aim of the present study was to explore healthy ageing in this group from the perspective of the leaders. Interviews with 20 leaders were subjected to qualitative content analysis. The findings gave rise to the overall theme ageing in dependence, which emerged from the following six categories: Supporting self-determination; Inaccessible activities after retirement; Signs of decline; Increased and specific needs for support and care; A non-question of gender; Aspects concerning the end of life and death. A prerequisite for healthy ageing in the case of people with ID is, according to the leaders, that they can live the life according to their preferences and make independent choices whilst at the same time receiving adequate support. With the shrinking of their social network after retirement, they become increasingly dependent on staff and leaders in the group home, who need to know what healthy ageing implies.

## 1. Introduction

People who have lived with intellectual disability (ID) since birth or early childhood constitute a vulnerable group when it comes to life changes such as ageing, because of the difficulties they may experience both in understanding their own need of support and care and in communicating with others about this need. The average lifespan of people with ID has significantly increased in the same way as for the population in general, as a result of medical and social progress [1]. The present generation of people with ID is the first to reach advanced average age [2,3]. Several studies [4,5,6] have found that an overwhelming majority of older people with ID experience problems concerning their physical and mental health, including cognitive decline. There is a risk of underestimating these problems, and the WHO [1] has drawn attention to the fact that people with ID who reach old age do not have the same opportunities for healthy ageing as the general population.

The European population is ageing, and, according to recent demographic forecasting, the number of Europeans over 65 will have risen to 148 million by 2060, representing an almost twofold increase compared with the 2010 figure of 87 million [7]. This constitutes a huge challenge to the financial systems, especially with regard to the sustainability of the health-care systems, and supporting active and healthy ageing is therefore crucial both for the senior citizens themselves and for society. The political goals concerning the older population are often discussed in terms of healthy ageing, which can be defined as a lifelong process with the potential to improve and maintain health. This includes physical, social and mental well-being, independence, good quality of life and successful transition from one stage of life to another [8]. The European Commission [7] has, with the implementation plan “European Innovation Partnership on Active and Healthy Ageing”, set the target of increasing the healthy lifespan of citizens within the EU by two years by 2020 and improving the health and quality of life of older people.

International research has indicated that ageing for people with ID differs from ageing for people of similar age in the general population in that it seems to set in earlier [9,10]. In a study about early ageing in people with Down syndrome (DS) the mean number of comorbidities was 5 in a study group of 31 adults with DS with a mean age of 51 years [11]. Ageing in people with ID could also be affected by long periods of institutionalization, long-term medication with psychotropic and long-term multiple health issues [1,12,13].

The WHO has called attention to the fact that older people with ID are a neglected group at risk of being treated unfairly when it comes to the provision of care and welfare (because they are being given lower priority than the older population in general) [1]. People with ID have the right to living conditions equal to those of the general population [14], and in Sweden the disability reform which came into effect in 1994 is designed to ensure equal rights for people with ID [15]. In the second half of 19th century and the first half of the 20th, Sweden established large institutions for people with disabilities and many lived their entire lives there. Towards the end of the 20th century deinstitutionalization became a trend in the Western world [16]. Several reforms were carried out with the design of furthering the integration into society of people with ID, and both daily activity centres and small group homes (where the flats are combined with shared areas and access to care and welfare staff) were established in Sweden. By the year 2000 almost all of the large institutions were closed [17] and people with disabilities had moved into flats of their own, some of which were located in small group homes with access to care staff around the clock.

In Sweden, people with ID can apply for support through the Act Concerning Support and Service for People with Certain Functional Impairments (LSS Act). This Act establishes that people with ID have the right to good living conditions, to be supported in their daily living and to exercise influence over how support is provided [15]. Support should be adapted to the needs of the individual and should strengthen the individual’s ability to live an independent life. Support in accordance with this Act can be given only to an individual who has personally (or with support from someone else) applied for it [15], and any decision regarding a measure under the LSS Act can be appealed to the County Administrative Court [18]. Intellectual disability services in Sweden in accordance with the LSS are financed by taxation and it is mandatory for the municipalities to provide these services. A reasonable fee may be charged for accommodation, recreation and cultural activities, but the individual must be left sufficient funds for personal needs [15]. The LSS includes the right to supported living and to participate at daily activity centres. The daily activity centres are intended to provide meaningful activities for those who are between 18 and 67, who are neither studying nor working. Activities are adapted to the capacity and interests of each participating individual.

Leaders for group homes and daily activity centres make strategic decisions concerning for instance financial priorities and the recruitment and training of staff, which are factors affecting the everyday lives of people with ID. Leaders can therefore greatly influence the conditions for a healthy ageing and for how the needs of support, services and care are met. Studies have shown the importance of leaders in organizations for people with ID. Leaders can have an indirect influence on outcomes for service users [19] through their leadership of the staff [20] and can contribute to an increase in social inclusion for persons with ID [21]. Another study showed that after supervision and training in active support for front-line managers, the residents with ID had an increased level of choice and adaptive behavior and a decreased level of depression [22]. Leaders for group homes or daily activity centres would seem to have a special role in the lives of people with ID. This requires that they have knowledge not only of the particular person’s specific needs but also of the ageing process and age-related issues in general. Therefore it is important to study leaders’ views of ageing in persons with ID. To the best of our knowledge there has been no previous scientific study published in English specifically focusing on leaders’ perspective on ageing for people with ID.

## 2. Aim

The aim of the study was to explore healthy ageing among people with ID from the perspective of leaders in intellectual disability services in Sweden.

## 3. Method

The design of the study was explorative and qualitative, and it was based on individual semi-structured interviews with leaders in the intellectual disability services for people with ID in a city in southern Sweden.

In this study the term leader is taken as being synonymous with manager and includes both front-line and middle-line leaders. The term intellectual disability service is taken to comprise group homes and daily activity centres for people with ID.

### 3.1. Setting

The current city is one of Sweden´s major cities and the municipality provides services for people with ID. Managers in the organization are organized at four levels, front-line leaders and middle-line leaders being the levels closest to the service users. The LSS Act includes supported living in a group home and support at a daily activity centre. Persons with different levels of ID were living together in a group home, 6‒8 persons with ID in each home, and they were 18 years or older.

Front-line leaders for the group homes in this study were responsible for two to three group homes, with service users and their direct care staff. Front-line leaders for the daily activity centres were in general responsible for two centres, with service users and their direct care staff, where staff supported persons with ID for a varying number of hours during daytime. Middle-line leaders for group homes or daily activity centres had a more strategic and operational responsibility.

### 3.2. Sample and participants

The inclusion criteria in this study were that the participants should have experience in leading services for people with ID who were 55 years or older living in a group home and/or participating at a daily activity centre. The managers at the city level with responsibility for all group homes and daily activity centres gave approval of the study. Then a letter containing information about the study and asking about willingness to participate was sent by one of the authors (PB) to all 12 middle-line leaders in the intellectual disability services (group homes and daily activity centres) in the city in question. Four of the 12 middle-line leaders agreed to participate. The middle-line leaders sent enquiries about participation to front-line leaders and 16 of them agreed to participate. The leaders decided themselves whether they met the inclusion criteria. It is not known why eight of the middle-line leaders declined to participate or how many leaders fulfilled the inclusion criteria but did not want to take part. Thus the study sample consisted of 20 leaders, four of them middle-line leaders and 16 of them front-line leaders. Fifteen of them managed group homes and five were leaders for daily activity centres. Five of the leaders were men and 15 were women.

### 3.3. Data collection

#### Interviews

The leaders who agreed to participate were contacted by e-mail in order to arrange a time for an individual interview. All of them chose their workplace for the interview. An interview guide with two main open questions was used, designed to provide a focus: 1. What does ageing well mean for a person with ID? 2. How do you perceive the needs of service, support and care in the ageing process for older persons with ID? The interviewees’ answers were followed up by probing questions which deepened the answers further. The interviews were recorded and transcribed verbatim.

### 3.4. Analysis

The interviews were subjected to inductive qualitative content analysis [23], and, in order to structure the analysis, the texts were stored in the software program NVivo (version NVivo10). The first step in the analysis was to read all the transcribed interviews thoroughly several times. The second step was to identify meaning units. The third step was to code the content of the meaning units, which involved a certain amount of interpretation, resulting in several categories. The fourth step was to go through the categories again and compare them with one another, whereby some were found to be superfluous. Interpretation of the content increased throughout the analytical process [23,24] and resulted in six categories from which one overall theme emerged. The theme is an expression of the underlying meaning revealed through codes and the core content of the categories [24].

The analyses were performed by the first author (MJ) and the trustworthiness of the findings was addressed by means of scrutiny of the data by all the authors together. The analyses were discussed at several meetings, and the codes, categories and theme were generated by consensus. Quotations from the interviews are used in order to illustrate the categories.

## 4. Ethical Considerations

The project was approved in 2013 by the managers responsible for health care and social welfare in the city in question, as well as by the Regional Ethical Review Board in Lund (Dnr 2013/83). All the participants received oral and written information to the effect that participation was voluntary, that they could discontinue participation at any time and that all information would be kept and treated confidentially. Written consent was given by all the participants before the interviews.

## 5. Findings

The overall theme “Ageing in dependence” emerged and was interpreted from six categories which were based on the interviewees’ statements: (1) Supporting self-determination; (2) Inaccessible activities after retirement; (3) Signs of decline; (4) Increased and specific needs for support and care; (5) A non-question of gender and (6) Aspects concerning the end of life and death.

### 5.1. Ageing in Dependence

The theme is intended to express that leaders stated that older people with ID living in a group home and attending a daily activity centre have lived in a situation where they depended on other people’s support, service and care since birth or early in life. The interviewed leaders found that a prerequisite for healthy ageing for persons with ID is the opportunity to live according to their preferences and to make independent decisions. At the same time, they depend on individualized degrees of support from staff in order to make the most of this opportunity. They may need, for instance, to be supported in acquiring skills in order to be as independent as possible. Or they may depend on staff for their daytime activities and, if they lack in verbal communication skills, on the staff’s ability to interpret their preferences. Older persons with ID depend on the ability of staff to recognize and notice signs of cognitive and physical decline and to discern the need for support due to these changes. Throughout the ageing process they depend on staff to discover any increase or change in need of assistive devices. According the leaders, there were no apparent gender-related differences in the ageing of older persons with ID or in their last days of life. They are also dependent on staff in order to handle grief in a healthy way.

#### 5.1.1. Supporting Self-determination

According to leaders, self-determination was an important prerequisite for ageing well. The leaders were of the opinion that ageing had the same significance for a person with ID as for anyone else: “ageing well” meant living an active life and continuing to develop skills as long as the person remained healthy and had the energy, but it also meant being able to decide to just take it easy at home and, for example, watch TV.

*I don’t see any difference at all in what it means to age well, whether you’ve got an intellectual disability or not. For me, ageing well means that you’ve always got the chance to do what you’re able to do on your own, to the extent that you want to.* (front-line leader)

The leaders also indicated that an important precondition for ageing well with ID and having a good life is that they should have the opportunity to make independent choices and, if necessary, receive adequate support for making such choices. This could be difficult, as many of them have lived most of their lives in large institutions where everyone had to follow routines and there was little chance of choosing independently. One example given was that of an old man who at first did not know how to make decisions about his own meals, but with the right support did learn how to do so.

*And now he does it himself. He cooks his food himself and he’s the one that decides what it’s going to be. He couldn’t do that before—even though he had the food in the fridge he couldn’t choose what to have. But now he can!* (front-line leader)

One leader spoke of how an older woman had developed confidence in her own capacity to express her wishes and complaints.

*Suddenly you’re visible, you’re important... I’m thinking of those dear old ladies, there was one of them that always wanted to do whatever everybody else wanted, never had a bad word to say about anybody, didn’t dare... but then suddenly there she was, bold as can be.* (front-line leader)

Ageing well for a person with ID could also require accessible information about the ageing process and what ageing means. The leaders thought there should be easy-to-read information about ageing, tailored for older persons with ID. It could be important for them to understand changes in the body and the cognitive capacity during the ageing process, as such knowledge could provide a sense of security. It is also crucial that the staff are attentive to signs of ageing and notice changes in a person’s daily condition. For example, older persons with reduced verbal communication skills depend heavily on the interpretive abilities of staff, who should be able to understand if, say, a person lacks energy but is unable to say so.

*Well, we’re always out to get the person to train walking, but in fact they can perhaps be allowed to go to some things in a wheelchair, because then they can save their energy for something they enjoy doing. I can see that we sometimes fall short there. You mean so well, you want their best*... (front-line leader)

Supporting self-determination sometimes poses a challenge for staff. It is part of life to make mistakes when making decisions, according to leaders, and they thought the staff should not, and indeed *cannot*, protect the older persons from all bad choices. People with ID have the right to choose junk food, to smoke and avoid exercise, just as anyone else. However, the staff should suggest healthy options. The leaders also thought that even if the older persons with ID chose not to participate in an activity (going to the theatre or cinema, for instance, or going out for a walk), it was important that they should be invited and should feel the choice was theirs whether to participate or not.

The person’s financial status can pose a limitation. Someone might want to go to London, for instance, but not be able to afford it. Here a leader mentioned that staff could help by offering accessible alternatives.

*A trip to Copenhagen could be as nice as a trip to London.* (front-line leader)

#### 5.1.2. Inaccessible Activities after Retirement

The fact that persons with ID now experience retirement presents a new situation, both for the persons themselves and for the staff at group homes. The leaders’ experience was that persons with ID reacted very individually to retirement. Some of them were looking forward to it; others felt considerable uncertainty about it. The leaders indicated that older persons with ID have reduced social networks and depend more on staff than do other older persons. There were very few of the older persons with ID whose parents were still alive. When they retired from the daily activity centre there was a further reduction in their social network and their opportunity for activities. This put pressure on staff at the group home to compensate for the reduction. There were recreation consultants in the municipality who could offer ideas for activities, but it was the task of staff at the group home to plan activities during daytime. However, leaders felt that the lack of staffing during daytime gave little opportunity for supporting the older persons’ activities.

All the leaders presented the same picture: activities for persons with ID provided by the municipality or voluntary organizations were usually inaccessible for older persons with ID. There were, for example, leisure activities for all ages, such as music evenings, film and dance. These were, however, with one exception, activities taking place in the evening, when the older persons with ID generally are too tired to participate even if they would like to. The interviewees pointed out that there was a risk that the older persons with ID´ were not being offered the activities and stimulation they needed, and they would like to see more possibilities for activities during the daytime.

*I can see that they’re a group that may very well have too few possibilities once the daily activity* centre’s *gone*. (middle-line leader)

The change in activities that retirement brought could be hard for some older persons with ID. If they had been to the daily activity centre for many years, it could be difficult for them to imagine that there was anything else they could do. The staff could talk about activities and show pictures of what to do at the group home during daytime. It was important to provide alternatives to the activities at the daily activity centre.

*It’s harder for our service users to visualize the possibilities, and they may need help in sorting out the alternatives*. (front-line leader)

It was unusual for the older person with ID to join an organization for leisure-time activities. Several of these activities were oriented towards IT technology and games, but few older persons with ID were able to seek information on the Internet or even showed an interest in it.

*But my old people aren’t very interested—it’s more younger people that think it’s fun*. (front-line leader)

The leaders also pointed out the need for more coordination between different group homes within the municipality, in order for them to be able to arrange joint activities during daytime for retired persons with ID—things like going to a café or going for a walk. However, the leaders considered that there is a risk that the persons with ID will just ask for activities they are used to, and therefore the staff must be open to new possibilities and encourage them to try something they have not done before.

*Now there’s the chance to do something completely new. I don’t quite know whether that’s an idea everyone shares—but I’d like it to be*. (middle-line leader)

#### 5.1.3. Signs of Decline

Signs of decline can be both physical and cognitive, and the ageing process was perceived by the leaders as being highly individual: some persons with ID were over 50 and still very active but in the case of others there was seriously marked ageing at the age of 40 to 50, and for some persons with severe disabilities or Down’s syndrome even earlier.

*When it comes to considerable disability, I can certainly say that when the person’s 40 we can see something that may be connected with ageing*. (front-line leader)

Signs of ageing can also come in the form of changes in personality: the older person may seem to be more confused or forgetful, become more tired or behave in a changed way, for instance. The leaders spoke of the difficulty of separating signs of dementia from normal ageing. Ageing could also manifest itself as a decrease in capacity—for example, a person might no longer have the energy for such long days at the daily activity centre or for the same activities as before. Often the older person chose calmer activities, which resulted in a changed social context.

*Your social sphere gets more reduced—you focus on a smaller area. There are things you used to do that you don’t do anymore because your body doesn’t let you. Perhaps you concentrate more on activities that you can do sitting down*. (front-line leader)

The leaders described several signs of ageing and gradual loss of abilities. The physical signs of ageing could be the same as for everyone else: greying hair, deeper wrinkles, reduced sense of balance, and difficulty in getting up from a chair or when walking. Hearing as well as vision could decline, and the body might become subject to contractures. A reduced appetite could result in a loss of weight. Body language could change and the person could become passive or aggressive, especially where pain could be suspected. The older person who finds it difficult to communicate and to handle pain is in need of support.

The leaders also expressed the opinion that older persons with ID are affected by diseases in the same way as the older population in general. Some diseases among older persons with ID mentioned were pneumonia, gastric and intestinal problems and urinary infections. Leaders assumed that a probable contributory factor in the case of these persons was that many of them use wheelchairs and did not move much. Other disorders mentioned were diabetes, depression, heart attack, stroke, chronic obstructive pulmonary disease and afflictions such as dizziness and incontinence. The leaders drew the conclusion that older persons with ID should have the opportunity to see a doctor at least once a year, especially in view of the fact that many of them have been on medication for years.

#### 5.1.4. Increased and Specific Needs for Support and Care

The leaders indicated that persons with ID who live in group homes already have enough resources at their disposal to meet their increased need for support as they grow older. As in the case of people in general, ageing and illness lead to an increased need for assistive technology to compensate for decreasing physical abilities.

*Well, I suppose it’s the same as for most of us: you usually need more support when you get older*. (front-line leader)

Due to the ageing process older persons with ID could come to need assistive devices, such as glasses, or hearing aids. Some assistive devices could be difficult to try out and it could therefore be necessary to have clearer instructions and more time, for example when visiting an optician or a physician. It is important that the staff at group homes and at daily activity centres are prepared to adapt the activities in accordance with the declining abilities of the ageing person with ID. The leaders indicated that the staff need to acquire more competence regarding health problems in order to more easily detect any need for assistive devices or increased care.

*Really it’s important, of course, for the staff to have all the competence that’s needed in the care of the elderly, because these people have the same needs as other people when they grow old*. (front-line leader)

The ageing process for persons with ID may be eased by staff being proactive and prepared for an increase in need of support. The staff might, for instance, adapt the environment and introduce assistive devices in advance, before disabilities caused by ageing begin to be noticed. One leader spoke about a man who began to find it difficult to walk. This man was very sensitive to changes. So they installed a ceiling hoist and allowed him to train with it in order for him to feel secure the day he really needed it. Thus, instead of starting with a mobile lift and then proceeding to a ceiling hoist, the staff chose the ceiling hoist directly, to spare him from an extra change.

*So I think it can be said that that’s really the big challenge: thinking ahead so as to make the change as comfortable as possible for the older persons, when they themselves perhaps haven’t got the ability to communicate what they want or don’t want*. (front-line leader)

A view that was commonly expressed was that the need for care increases in areas such as assistance with hygiene, clothing, cleaning, transportation and meals. The leaders also indicated that persons with ID could need more support to compensate for the gradual loss of cognitive functions.

*And so there comes a day where things sort of just get confused... You set up the first activity and then you maybe go to the blackboard again, or to the pictures, and then the person can see what the next thing is, and then things go all right.... That’s the way it is, the cognitive support. You have to try to understand where, sort of, the person is now*. (front-line leader)

Many persons with ID are accustomed to using cognitive aids (e.g., pictograms or concrete objects) due to their disabilities. However, the leaders had found that with increasing age the understanding of such aids could change: the support was the same as before but the person with ID responded differently.

*You see, the person’s not the same as usual. We give roughly the same support but the response isn’t the same. Then when we’ve tried to put two and two together it may strike us that it’s not the illness as such. “Yes, but what age is he [or she]?” And that gives us something to think about*. (front-line leader)

The leaders also pointed out that there was an increased need of support for older persons with ID when signs of dementia appeared.

*She does a lot of things—she gets confused and runs out on to the road without putting any clothes on. Because of this, I’ve had to make it a rule that there are to be more staff so that somebody is always near her to prevent that sort of thing from happening, and extra supervision during the night*. (middle-line leader)

Leaders pointed out that it is important that all changes should be documented, particularly if the person with ID cannot communicate verbally, so that new staff has access to information about the person’s needs and their ageing process. It is furthermore important that the staff should be responsive, able to interpret the person’s expression of needs and wishes by non-verbal signals—by means, for instance, of behavior, facial expression, eye movement or body language. The need for support, service and care has to be met regardless of age—and the leaders indeed pointed out that such need varies more because of individual differences than because of ageing itself.

*I look at it this way: you attend to the need regardless of the age factor. I don’t feel it’s specifically connected with ageing—it’s more a question of the situation on the particular day or of how the person feels in general*. (front-line leader)

The leaders noted that one problem in responding to and caring for older persons with ID today is that many of them have lived in large institutions and thus have a limited documented life-history, as such information was not documented in those settings. If the verbal communication skills of the older person are limited and there is little or no documentation, the staff can find it difficult to properly understand and meet the person’s needs. For example, if the person has a dementia disease, the lack of understanding may create anxiety and frustration. The staff groups are mostly younger persons who do not know what life was like at the large institutions or what experiences the old persons might have had there.

*It’s enormously difficult for us who are working today to imagine what it was like at the institutions. Which means we can’t grasp what’s going on, even though it may be a question of something that’s vividly present to the person*. (front-line leader)

#### 5.1.5. A Non-question of Gender

The interviews with the leaders did not indicate that there were any differences between men and women with regard to ageing. Individual needs and preferences govern what activities and assistance were offered by the staff. A common response among the leaders when considering differences between men and women with respect to the need of support, service and care, was that they had not given it much thought. The needs were met on an individual basis regardless of gender.

*There are big differences between one person and another but, no; I don’t think there’s any difference between men and women in this respect.* (middle-line leader)

However, the leaders did indicate that, even though the *need for* support, service and care varied on an individual basis and not according to gender, there might in fact be gender-related differences in the support *actually given*. Persons who are more discreet and quiet than others can have difficulty in asserting their needs, and by tradition this may indeed be gender-related. Thus women may find it more difficult to make their voices heard; and older women run the risk of having their needs less adequately met than older men, if the staff are not attentive to the problem.

*But I think it’s perhaps expecting a bit more of staff, that there should be equal support and service. Yes, I really do think so*. (front-line leader)

The leaders were careful to emphasize that their experience involved so few individuals that they did not think it would be possible to draw any general conclusions from it. When the staff at one group home gathered statistical data about the activities they provided, they noticed that men were offered individual activities while women were offered more activities together with others.

*We offer more group activities to women than we do to men. The men are offered more individual activities. But the women are satisfied—whilst the men are less satisfied with group activities*. (front-line leader)

#### 5.1.6. Aspects Concerning the End of Life and Death

According to leaders, persons with ID handle issues of death and the last days of life very individually. Some engaged in the topic, while others avoided it.

*There are many who talk a lot about death: “Daddy’s dead!”—and they visit the grave at the churchyard... And there are others who simply don’t want to broach the subject at all*. (front-line leader)

The leaders thought that it was best for persons with ID approaching the end of life to stay on at the group home where they were familiar with the environment and staff, and thus felt secure. Due to this, it has become necessary for the staff to expand their knowledge of palliative care in order to attend to dying persons with ID. Staff groups in intellectual disability services are often not accustomed to caring for persons at the end of life, which can create both anxiety and sadness. It may be necessary for staff to be supported in coping with their own and the older person’s feelings.

*They need to be able to handle the person’s anxiety and they need to be prepared for whatever the future may hold—the particular development of the illness, for instance, and what this involves*. (front-line leader)

The leaders observed that since the number of persons with ID getting old today is increasing, they can come to live at the group home for a very long time, whereby the staff and the persons can become like a family, and deep grief can be felt by all when someone passes away. It is difficult to know whether a person with severe ID perceives that someone had passed away, but in persons with mild to moderate ID a death may trigger a grief reaction.

*There can be strong reactions as grief, and anger, as well as fear of dying themselves. It can bring back memories of, say, Mummy who died*. (front-line leader)

There were no general guidelines as to how staff at group homes or daily activity centres should handle someone’s death, but usually they have a quiet moment together and light a candle beside a portrait of the deceased. Some persons with ID or staff may go to the funeral.

## 6. Discussion

The main finding was that leaders considered that healthy ageing for persons with ID implied living according to their preferences and making independent choices, with support if necessary. Leisure activities provided by the municipality or voluntary organizations, were largely inaccessible for older persons with ID who no longer went to a daily activity centre, since such activities mostly took place in the evening. Signs of ageing were both physical and cognitive, but the need of support depended more on the individual than on age as such. Further, the leaders perceived no differences between men and women when it came to the ageing process, nor when it came to the need of support during ageing. Leaders were of the opinion that the best thing for the older person with ID approaching the end of life was to stay in the group home.

During the last five years there has been a marked increase in knowledge about ageing with ID, and most of the literature is concerned with the higher rates of diseases and health problems among older people with ID as compared with older people in general [5,6]. The aim of the present study is to provide knowledge about healthy ageing, the latter being a policy term used by the WHO and the European Commission. The increased rate of survival in the ageing population with ID means that more people are living longer with functional health problems and illness. Now is the time, therefore, to concentrate more on healthy ageing, which is sparsely represented in the literature. Healthy ageing is going to be one of the most important concerns for European welfare systems in the coming decades, with specific focus on maintaining, restoring and improving citizens’ functional capacity [25]. The focus of the present study is on the knowledge held by leaders in intellectual disability services in Sweden with respect to new or increased needs during the course of ageing in persons with ID. This knowledge is critical with regard to supporting staff in the provision of person-centered service for healthy ageing. Previous research has clearly indicated that the quality of management in the intellectual disability services is an important determinant of staff practice [20].

Healthy ageing is defined by the WHO as “the process of developing and maintaining the functional ability that enables wellbeing in older age”. Functional ability is determined both by the individual’s intrinsic capacity (physical and mental) and by environmental factors, and also by the interaction between the two [26]. The WHO gives many examples of environmental factors, including social protection, social facilities, health- and long-term care, relationships, family, and care staff. Such factors, for example support from care staff, have an indispensable role to play in compensating for intellectual disability and promoting healthy ageing. The leaders’ view of healthy ageing is expressed through the category Supporting self-determination, that means with the right support older persons with ID can make independent choices with a higher degree of self-determination, while depending on staff when it comes to the acquisition of greater self-reliance. The staff, in turn, may require the support of the leader in the task of supporting the older persons in living according to their preferences. The task is a challenging one, because older persons with ID often find it difficult to communicate their needs and therefore are dependent on the ability of staff to interpret non-verbal expression. Leaders and staff also need to bear in mind that the majority of persons with ID who are now elderly have grown up in an institution (or at least lived a long time in one) [27] and can experience difficulties in expressing their whishes and to make choices.

Signs of decline (e.g., diminished mobility or cognitive capacity) in persons with ID were considered by the leaders as being no different from those in the rest of the population. Ageing was most importantly highly individual, but it can come earlier in persons with ID, as has been shown by previous research [10]. Retirement from organized daytime activity constitutes a watershed in respect of ageing, though signs of ageing can be difficult to detect in the case of some people with ID who age earlier [9]. Ageing which begins long before retirement and proceeds slowly can escape notice, whereby needs connected with ageing may remain unfulfilled. Cognitive changes can remain undetected until there is a crucial time when it becomes evident that the person requires more support or a different form of support. Kåhlin [28] found that staff at a group home regarded the ageing of persons with ID as being mainly a bodily and medical process. In our own study the psychosocial aspects of ageing did not emerge very clearly. Covelli et al. [29] point out that as a consequence of the small amount of research which has been done on social and environmental factors, decision-makers and leaders do not become aware of them and as a result the needs of the service users remain unseen and thus unfulfilled.

It is clear from the leaders’ answers that there were no organized activities for older persons with ID at group homes after retirement from daily activity centres. The leaders experienced it as problematic to compensate with activities for those who needed it, because of low staffing during daytime at group homes, but at the same time they were aware of the fact that the older person’s social network might consist entirely of staff. It is a delicate situation, partly because of the high turnover of staff and partly because of the variation in the degree of competence possessed by staff when it comes to providing support for activities. The older persons with ID are vulnerable, and there is a risk that many of them miss the stimulation and companionship offered by the daily activity centre. Graham et al. [30] and Chou et al. [22] point out that methods such as active support or training of active support can raise the staff’s level of awareness concerning activities and lead to there being a greater choice of activities for the person who remains at the group home during the day. Daytime activities provided by the municipality, for example by several group homes collaborating, could reduce the isolation of older persons with ID and enable them to meet other people of their own age. At the same time there must be staff at the group home during the day, because a person must be able to choose whether to make the effort to participate in activities elsewhere or instead stay at home, depending on preferences on a particular day.

The leaders did not see any differences between men and women with regard to ageing and the need for support. They had, in fact, not previously given any thought to the matter, and this, they said, might be because they had experience with so few older people with ID. Not seeing the person as a man or a woman may be an expression of the need to see him or her as an individual, or it may instead be the case that the intellectual disability overshadows other characteristics of the person. Even in feminist research stretching over several decades, the special position of women with ID has been largely ignored [31]. Research has shown that normative conceptions govern what is to be regarded as masculine or as feminine. Gender plays a part in the formation of social roles, where women are regarded as subordinate. In the present study, a group home kept statistics about activities offered by staff, and found that women were offered group activities whilst men were offered individual ones. The men were not satisfied with group activities, while the women were, but in the light of Barron’s study [31] it would seem appropriate to ask whether in the women’s case it was not so much an expression of real choice as an expression of subordination and a desire not to put the staff to any trouble. Thus it is not just disability and age that have to be taken into consideration, but also gender. The concept of intersectionality can aid in the understanding of how a person’s several social identities interact [32]. The leaders in intellectual disability services usually have a university level of education, and often in social sciences. It is therefore a little surprising that not one of the leaders ever made reference to such terms as “double disadvantage” (ID and ageism) or “triple disadvantage” (ID, ageism and being a woman) [31]. It is important that future research be based on intersectionality, in order to provide a better understanding of healthy ageing in the case of persons with ID.

According to leaders, the older persons with ID had different ways to handle thoughts on death, from engaging in the topic, to not speaking about death at all. The leaders thought it might be better for the person approaching the end of life to remain at the group home and being cared for by the staff there, rather than moving somewhere else to receive palliative care. At the same time, staffs at group homes seldom have training in palliative care, and they need instruction and supervision if they are to be able to provide such care and ensure that the older persons end their lives with dignity. Wiese et al. [33] found that even if staff thought a person with ID had a right to information about death, they nevertheless had little practical experience in conveying such information and might indeed try to avoid the subject in their encounter with the person. Furthermore staff referred to the problem of limited capacity to understand death as an abstract phenomenon by people with ID. However, it is possible that the staff underestimate this capacity. It emerged from the interviews in the present study that there were opportunities for talking about death if the older person wanted to, for instance before or after a visit to the cemetery. Tuffrey-Wijne and Rose [34] suggest that service managers should make sure that the staff are trained in different questions about death and bereavement, as a means of improving their ability to communicate with and support the people with ID.

The leaders called for information about physical and cognitive changes associated with ageing which the people themselves could understand. Tuffrey-Wijne et al. [35] have drawn up guidelines for deciding whether information about serious illness and death should be given to the service user and the European Association for Palliative Care (EAPC) has published a White Paper on palliative care for people with ID in Europe [36]. According to leaders in this study there was a need for giving information about ageing to the older people with ID, and we suggest that a guideline about ageing and the changes ageing brings could be developed, as also about the question of if, when and how information could be given to the older person with ID.

### Methodological considerations

The transferability of the findings from this study needs to be carefully considered in light of the study’s limitations. One is that the participants in the current study all worked in the same municipality in an urban area. This could affect the findings, compared to a study sample from organizations in different communities, including rural areas. The findings might also have been affected if private providers of intellectual disability services had been included in this study. The leaders decided themselves if they met the inclusion criteria. Maybe the findings would have been different if we had started the data collection by identifying group homes were older persons with ID lived, and sent a request to the responsible leaders. An ongoing major reorganization of the intellectual disability services in the current city may also have affected the opportunities to participate.

The decision to interview the leaders in intellectual disability services instead of interviewing the older persons with ID themselves was based on the assumption that the latter constitute a vulnerable, frail and marginalized group [37]. The qualitative design based on interviews with leaders makes for the acquisition of relevant knowledge about ageing at the same time as it protects the older persons’ autonomy and integrity. The analysis of the interviews was performed by the first author but involved discussions by all the authors at a number of meetings, whereby changes were made. Certain categories were removed, others became more clearly defined. The inclusion of quite a large number of excerpts from the interviews is intended to illustrate the categories and make it easier for the reader to judge the credibility of the analysis. Occasionally the leaders may have made reference to someone who was under 55, in spite of the fact that we emphasized both in the information which was given prior to the study and during the interviews themselves, that the study concerned people who were 55 or more. The interviews were conducted at the leaders’ workplaces but in rooms which were not within sight or earshot of the service users. It was important that the latter should not be disturbed and that there should be no risk of their hearing anything which might upset them [37].

## 7. Conclusions

In this study, the leaders in group homes and daily activity centres for people with ID regarded healthy ageing for older persons with ID as being a question of being able make independent choices and live according to their preferences. When people with ID retire from daily activity centres they lose not only the activities but also the social network which they have had for many years, and there is a risk of being isolated and increasingly dependent on the staff at the group home, who in many cases become their entire network. The results indicate that there is a lack of organizational arrangements to meet the older service users´ need of accessible activities. There may also be a need for policy documents with regard to the natural ageing process and healthy ageing, and further education among leaders. This is in order for the leaders to be able to improve the support for older persons with ID and ensure that they enjoy an active and meaningful old age on their own terms, from both a physical, psychosocial and existential perspectives.
